# IDLaS-NL – A platform for running customized studies on individual differences in Dutch language skills via the Internet

**DOI:** 10.3758/s13428-023-02156-8

**Published:** 2023-09-25

**Authors:** Florian Hintz, Olha Shkaravska, Marjolijn Dijkhuis, Vera van ‘t Hoff, Milou Huijsmans, Robert C. A. van Dongen, Levi A. B. Voeteé, Paul Trilsbeek, James M. McQueen, Antje S. Meyer

**Affiliations:** 1 Max Planck Institute for Psycholinguistics, P.O. Box 310, Nijmegen, 6500 AH The Netherlands; 2 Deutscher Sprachatlas, Philipps University, Marburg, Germany; 3 Radboud University, Nijmegen, The Netherlands

**Keywords:** Web-based testing, Individual differences, Language skills

## Abstract

We introduce the Individual Differences in Language Skills (IDLaS-NL) web platform, which enables users to run studies on individual differences in Dutch language skills via the Internet. IDLaS-NL consists of 35 behavioral tests, previously validated in participants aged between 18 and 30 years. The platform provides an intuitive graphical interface for users to select the tests they wish to include in their research, to divide these tests into different sessions and to determine their order. Moreover, for standardized administration the platform provides an application (an emulated browser) wherein the tests are run. Results can be retrieved by mouse click in the graphical interface and are provided as CSV file output via e-mail. Similarly, the graphical interface enables researchers to modify and delete their study configurations. IDLaS-NL is intended for researchers, clinicians, educators and in general anyone conducting fundamental research into language and general cognitive skills; it is not intended for diagnostic purposes. All platform services are free of charge. Here, we provide a description of its workings as well as instructions for using the platform. The IDLaS-NL platform can be accessed at www.mpi.nl/idlas-nl.

## Introduction

Over the past decade, psycholinguistics has seen a growing interest in research on individual differences. Researchers have begun to acknowledge that comprehensive models must accommodate variability between language users, rather than focusing entirely on the average or group behavior. Indeed, according to theoretical views, individual-differences studies provide a powerful source of evidence bearing on key issues in the language sciences, such as the architecture of the language system and the mechanisms supporting language use (Kidd et al., 2018; Siegelman et al., 2017). As a result of this shift in thinking, the number of studies using individual-differences approaches has been steadily increasing (e.g., Dabrowska, 2018; Engelhardt et al., 2017; Favier et al., 2021; Isbilen et al., 2022; James et al., 2018; Johns et al., 2018; Li et al., 2019; McMurray et al., 2010; Schmidtke et al., 2018).

In spite of this positive trend, there are (at least) three reasons that might hold back labs from running studies on individual differences in language skills. The first reason relates to individual differences being best assessed using a multitude of tests for measuring the same underlying psychological construct (Miyake et al., 2000). Just as the vast majority of behavioral tests, tests measuring language skills suffer from the so-called ‘task impurity’ problem. Task impurity refers to the fact that performance on any single behavioral test is likely influenced by a multitude of skills (e.g., a speeded lexical decision task involves word recognition/lexical access *and* a speeded motor response). Thus, using a single test to gauge a psychological construct is likely to conflate the skill of interest with other skills. By using multiple tests that tap into the same underlying psychological construct but vary in their surface structure and/or response variable, researchers can apply statistical techniques that partial out unwanted variance and extract variance that is shared across the tests and reflect the skill of interest.

Second, since using language inherently involves general cognitive skills (e.g., non-verbal processing speed, Hintz et al., 2020b; Huettig & Janse, 2016; working memory, Baddeley, 2012; non-verbal reasoning, cf. Deary et al., 2007), variability in language skills should be characterized in concert with variability in general cognitive skills. This necessitates the inclusion of tests measuring the respective general cognitive skills involved in the language task(s) of interest.

The third reason is that individual-differences studies require large numbers of participants to achieve sufficient statistical power. As described by Schönbrodt and Perugini (2013), simple correlation coefficients stabilize at a sample size of 161 participants. As pointed out by Brysbaert (2019), for an effect size of *d* = .4 (corresponding to a correlation of *r* = .2, *p* < 0.05, two-tailed), 194 data pairs are required, which is much more than the number of participants typically tested for studies using factorial designs.

Taken together, large numbers of participants who each complete large numbers of tests amount to participant fees and testing time that many labs cannot afford. A critical bottleneck also concerns the man/woman power required for test administration and data pre-processing, in particular if the collected data involve manual transcription and annotation (but see Stark et al., 2023, for an alternative solution).

An alternative to lab-based test administration is remote testing via the Internet, which is becoming increasingly popular. The availability of fast, flat-rate Internet connections and affordable computer hardware for home use, as well as the host of open-source and commercial solutions for psychological testing have motivated many researchers to move their studies online. Indeed, as reported by Anwyl-Irvine et al. (2021), the number of papers tracked by Web of Science with the keywords ‘MTurk’ or ‘Mechanical Turk’ (Amazon’s platform for accessing online participants) increased from 121 publications in 2013 to 642 in 2018. Online testing speeds up data collection while yielding more diverse participant samples than typically seen in lab-based testing (Garcia et al., 2022). Moreover, systematic comparisons of lab-based and online test administration (e.g., Cheung & Rensvold, 2002; Garcia et al., 2022; Germine et al., 2012; Ruiz et al., 2019; Cieciuch and Davidov, 2015, for a tutorial) support the notion of measurement invariance. That is, while the absolute numbers may differ (e.g., reaction times recorded in a web experiment are likely longer than those recorded in the lab due to inferior hardware and software at home), the differences between condition means in an experiment and the relationships between scores on different tests has been shown to be comparable (Hintz et al., in prep.). Given these advantages, online testing seems the perfect solution for alleviating some of the challenges properly powered and well-designed individual differences studies face.

Indeed, there are a number of existing commercial and open-access test batteries for assessing individual differences in language skills and skills related to language processing via the internet (e.g., Human Cognition Project, Morrison et al., 2015; PEBL, Mueller & Piper, 2014; Alberta Language Function Assessment Battery, Westbury 2006; ACS, Feenstra et al., 2018; PsyToolbox, Stoet, 2017)—some of which can be turned into customized batteries. However, to our knowledge, there is no solution yet that accommodates all three of the issues outlined above: (1) offering a test battery with multiple tests per psychological construct, (2) including tests measuring language *and* general cognitive skills involved in language processing, and (3) offering a comprehensive and user-friendly system for running these tests via the internet.

The present paper introduces the Individual Differences in Language Skills (IDLaS-NL) web platform that allows researchers to run customized studies on individual differences in language skills via the internet. The target language is Dutch, and the target skill sets are word and sentence production and spoken word and sentence comprehension. We make available a set of 35 Dutch behavioral tests that were previously validated in participants aged between 18 and 30 years of age. The tests are hosted on servers at the Max Planck Institute for Psycholinguistics. Selections of the tests can be combined into studies, which may consist of one or multiple sessions—depending on the researchers’ needs. Studies can be created, managed, and deleted using an intuitive graphical user interface. We also make available a dedicated application (an emulated *browser*) wherein the studies are run to facilitate standardized test administration (i.e., each participant should use the same application). Upon start, the application takes up the full computer screen and thus reduces the likelihood of participants running other, potentially resource-consuming applications on the side. Each test has its own online database where the collected test data are stored. Results can be retrieved by mouse click within the graphical user interface. Item-level outputs, for some tests complemented with aggregated scores by participants, are made available via e-mail. All services on the IDLaS-NL platform are free of charge.

In the remainder of this article, we provide more information about the tests and their validation. We introduce the elements of the web platform, including the IDLaS-NL website with useful information for researchers, the graphical user interface for creating, managing, and deleting studies, and the test application. Finally, we describe how to use the platform and give some practical recommendations.

## The individual differences in language skills test battery

Funded by the ‘Language in Interaction consortium’ (https://www.languageininteraction.nl/), IDLaS-NL was developed by researchers based at the Max Planck Institute for Psycholinguistics and at the Donders Institute for Brain, Cognition and Behavior between 2017 and 2022. The main focus of our research program was to capture and explain variability in linguistic processing skills, more precisely variability in spoken word- and sentence processing. Our target group was native or near-native users of Dutch between 18 and 30 years of age.

To decide which tests to include in the test battery, we relied on a working model (McQueen & Meyer, 2019), which specified how the skills sets might be organized. As a broad theoretical perspective, we adopted the received view in the literature and assumed that speaking and listening involve overlapping, but not identical skills and therefore must be assessed separately, both on the word and the sentence level. Thus, our battery includes tests of linguistic processing in these four areas. We also assumed that speaking and listening skills depend on linguistic experience, which manifests in knowledge about the words and grammar of the language. Thus, the battery includes a linguistic experience component assessing such knowledge. Finally, performance in linguistic tasks is likely to be affected by domain-general cognitive skills, in particular processing speed, working memory and non-verbal reasoning, which must also be assessed. In sum, the tests in the battery measure the following eight psychological constructs: linguistic experience (six tests), non-verbal processing speed (five tests), working memory (two tests), non-verbal reasoning, word production (four tests), sentence production (four tests), word comprehension (four tests), and sentence comprehension (four tests). To address the task-impurity problem, all constructs, except for non-verbal reasoning, are assessed with multiple tests. An important practical requirement for the selection was that the tests are suitable for administration via the internet.

To develop the battery, we conducted several pilot studies featuring parts of the tests (Brysbaert et al., 2020; Hintz et al., 2020b; Kapteijns & Hintz, 2021; Jongman et al., 2021) as well as one large pilot study that involved testing 112 participants twice on all battery tests, with approximately 1 months’ time in between, for assessing test–retest reliability (Hintz et al., 2020a, 2022).

The final version of the test battery consists of 29 tests, which have been validated for young Dutch speakers in the Netherlands. Note that there are six additional tests on the web platform (see below), which are not part of the battery we developed but complement the psychological constructs we assessed. Note also that in our main study (Hintz et al., in prep.), we also administered the Peabody Picture Vocabulary Test (Dunn & Dunn, 1997; Schlichting, 2005) and Raven’s Advanced Progressive Matrices (Raven et al., 1998) as these tests are often regarded as the gold-standard for assessing receptive vocabulary size and non-verbal reasoning, respectively. However, these tests are copyright protected and are therefore not made available (but see ‘Additional tests’ for open-access alternatives). Table 1 lists all tests, along with brief task descriptions. For more extensive descriptions, including materials, procedure, and descriptive statistics based on a of sample of 748 participants, see Hintz et al. (in prep.).

**Table 1:** Overview of behavioral tests available on the IDLaS-NL platform

Domain	Task	Duration	Task description	Performance Indicator	Source
Linguistic experience	Stairs4Words (receptive vocabulary)	7 min	Adaptive vocabulary test based on a Yes/No decision task	Accuracy	-
	Antonym production (productive vocabulary)	5 min	Participants hear a spoken word and are instructed to produce its antonym	Accuracy	(Mainz et al., 2017)
	Idiom recognition	3 min	Participants select the correct meaning for an idiomatic expression among four alternatives	Accuracy	-
	Spelling test	5 min	Participants identify incorrectly spelled words in a list of 60 words (half of which are spelled incorrectly)	Accuracy	-
	Author recognition test	5 min	Participants identify fictional writers in a list of 132 names (90 are writers)	Accuracy	(Brysbaert et al., 2020)
	Prescriptive grammar test	10 min	Participants carry out grammaticality judgements on spoken sentences featuring morpho-syntactic constructions known to be difficult for adult speakers of Dutch (e.g., ik vs. mij, als vs. dan, ze vs. hun)	Accuracy	(Favier et al., 2021; Hubers et al., 2016)
Processing speed	Auditory simple reaction time test	3 min	Participants respond as quickly as possible to the presentation of a beep by pressing the space bar	Reaction time	(Hintz et al., 2020b)
	Auditory choice reaction time test	4 min	Participants respond as quickly as possible to the presentation of one of two beeps (high or low) by pressing one of two buttons	Reaction time	(Hintz et al., 2020b)
	Letter comparison test	5 min	Participants indicate as quickly as possible whether two letter strings are identical by pressing one of two buttons	Reaction time	(Hintz et al., 2020b)
	Visual simple reaction time test	3 min	Participants respond as quickly as possible to the presentation of a geometrical shape by pressing the space bar	Reaction time	(Hintz et al., 2020b)
	Visual choice reaction time test	4 min	Participants respond as quickly as possible to the presentation of one of two geometrical shapes by pressing one of two buttons	Reaction time	(Hintz et al., 2020b)
Working memory	Digit span	7 min	Participants are instructed to recall sequences of digits in the order they were encountered (forward version) or in the reversed order (backward version)	Accuracy	(Wechsler, 2004)
	Corsi block clicking	7 min	Participants are instructed to recall sequences of identical spatially separated blocks by clicking on them in the order they were encountered (forward version) and in the reversed order (backward version)	Accuracy	(Berch et al., 1998)
Word production	Picture naming	7 min	Participants name pictures whose names vary in word frequency as quickly as possible	Reaction time	(Hintz et al., 2020a)
	Rapid automatized naming	7 min	Participants are familiarized with four sets of five objects (colored line drawings of common objects (e.g., horse, mouth) whose names vary orthogonally in frequency and neighborhood density (operationalized as the number of words that can be obtained by adding, deleting, and substituting one phoneme in the target word). Each object set is arranged in an array consisting of five rows of six objects. Participants are instructed to name all objects in the array as quickly as possible	Reaction time	(Araújo et al., 2021)
	Verbal fluency	5 min	Participants name as many words as possible belonging to pre-specified categories (semantic part) and starting with letters provided ahead of time (phonological version) within 1 min	Accuracy	(Shao et al., 2014)
	Maximal speech rate	3 min	Participants are instructed to name the months of the year as quickly as possible	Reaction time	-
Sentence production	Phrase generation	10 min	Participants are familiarized with a set of 16 common objects. These objects are presented in noun/adjectival phrases of increasing difficulty, which participants are instructed to name as quickly as possible	Reaction time	-
	Sentence generation (structured)	12 min	Participants describe scenes depicting transitive actions (intransitives serve as fillers) as quickly as possible using pre-specified verbal material. Color-coding of the characters in the scenes coerces the production of active and passive sentences	Reaction time	(Menenti et al., 2011)
	Sentence generation (unstructured)	10 min	Participants describe scenes depicting transitive and intransitive actions and are free in their choice of words and syntactic structure/voice	Reaction time	(Konopka & Meyer, 2014)
	Spontaneous speech	4 min	Participants speak freely for one minute about three topics provided ahead of time	Reaction time/Accuracy	-
Word comprehension	Monitoring in noise in non-word lists	10 min	Participants monitor lists of non-words, presented in increasing levels of noise, for the occurrence of probe non-words, presented in the clear at the beginning of a trial	Accuracy	-
	Rhyme judgement	5 min	Participants are presented with two non-words in succession and are instructed to judge as quickly as possible whether both non-words rhyme	Reaction time	(McQueen, 1993)
	Auditory lexical decision	7 min	Participants judge the lexicality status of an auditorily presented target word as quickly as possible	Reaction time	(Hintz et al., 2020a, b)
	Semantic categorization	5 min	Participants judge as quickly as possible whether an auditorily presented target word belongs to a pre-specified semantic category	Reaction time	-
Sentence comprehension	Monitoring in noise in sentences	10 min	Participants monitor predictable and non-predictable sentences, presented in increasing levels of noise, for the occurrence of probe words, presented in the clear at the beginning of a trial	Accuracy	-
	Verb semantics activation during sentence comprehension	7 min	Participants are presented with two objects on the computer screen and a spoken sentence containing a target noun, which refers to one of the two objects. In half of the sentences, the target is predictable based on verb semantics. Participants indicate by button press which of the two objects is referred to in the sentence. On predictable trials, participants may respond before target noun onset	Reaction time	(Hintz et al., 2017)
	Gender cue identification	10 min	Participants indicate for 80 objects whether their names are de- or het-nouns (both common gender in English). *It is advised to run this test preceding the test ‘Gender cue activation during sentence comprehension’*. *Both are counted as one test*	Accuracy	*-*
	Gender cue activation during sentence comprehension	10 min	Participants are presented with two objects (the same as in the Gender cue identification test) on the computer screen and a spoken sentence containing a target noun, which refers to one of the two objects. In half of the sentences, the target is predictable based on a determiner expressing the grammatical gender of the target noun. Participants indicate by button press which of the two objects is referred to in the sentence. On predictable trials, participants may respond before target noun onset. *It is advised to run this test following the test ‘Gender cue identification’. Both are counted as one test*	Reaction time	(Huettig & Janse, 2016)
	Self-paced reading	5 min	Participants read sentences of varying syntactic complexity in a self-paced fashion	Reaction time	(Uddén et al., 2022)
Extra tests	Dutch auditory & image vocabulary test	12 min	Participants hear a spoken word and select the picture associated with its meaning among four alternatives	Accuracy	(Bousard & Brysbaert, 2021)
	Matrix reasoning test	8 min	Participants indicate which of four possible shapes completes a matrix of geometric patterns	Accuracy	(Chierchia et al., 2019)
	BIG 5 personality traits	5 min	Participants rate personality statements	Qualitative	(Denissen et al., 2020)
	Receptive vocabulary test (multiple choice)	7 min	Participants read target words (varying in difficulty) and select the correct meaning among four written alternatives	Accuracy	(Vander Beken & Brysbaert, 2018)
Extra tests production	One-minute reading test	3 min	Participants read as many words (increasing in difficulty) as possible within 1 min	Reaction time	(Callens et al., 2012)
	Story reading test	5 min	Participants read as much as possible of a story within 3 min	Reaction time	(Rouweler et al., 2020)

### Additional tests

In addition to the tests that were part of our battery development efforts, we make available six tests that were previously developed and validated by researchers at Dutch, Belgian, English and US universities. The materials for these tests are free for use in scientific research, and we used them for our own implementations of the test. The tests are listed under ‘Extra tests’ and ‘Extra tests production’, respectively, in the graphical user interface and measure the following psychological constructs: *Linguistic experience* (piloted in Flemish speakers; i.e., receptive vocabulary size*,* ‘Dutch Auditory & Image Vocabulary Test’, Bousard & Brysbaert, 2021; ‘Receptive vocabulary test (multiple choice)’, Vander Beken and Brysbaert, 2018), *non-verbal reasoning* (‘Matrix reasoning test’, Chierchia et al., 2019), *personality traits* (‘BIG 5 personality traits’, Denissen et al., 2020), *word-reading* (piloted in Flemish speakers; ‘One Minute Reading Test’, Callens et al., 2012), and *story-reading* (piloted in Flemish speakers; ‘Story Reading Test’, Rouweler et al., 2020). We included these tests as alternatives for copyright-protected tests we previously used to assess receptive vocabulary size and non-verbal reasoning. The reading tests were included to capture the mediating influence that reading ability may have on spoken language processing (e.g., Huettig & Pickering, 2019). See Table 1 for brief descriptions of the additional tasks; for more information on the development and validation, see the paper(s) associated with each test.

## Technical details of the IDLaS-NL web platform

### Website

The website www.mpi.nl/idlas-nl provides a brief introduction to and overview of the IDLaS-NL platform. It describes the platform’s most important features, provides some scientific background, a user manual, and an FAQ page. The website links through (link to be found in the ‘User manual’ section) to the graphical user interface where studies can be created, edited and deleted, and where results can be retrieved. The website’s default language is English, but Dutch is available too.

### Graphical user interface

The IDLaS-NL graphical user interface is implemented in PHP. The landing page provides a brief overview of the platform’s functionality. To be able to access the subsequent page, users must provide an e-mail address, accept the ‘Terms of Use’ and type in the name of the Dutch city where the Max Planck Institute for Psycholinguistics is located. This CAPTCHA is necessary to prevent bots from automatically creating large numbers of studies. When users select the tests they wish to include in their research, determine the number of test sessions and the order of tests within the session(s), their configurations are stored in a database. This study configuration is associated with the provided e-mail address and a generated researcher key. Moreover, a unique study key is generated for the configuration and stored alongside the other information (see below for a more extensive description).

### Framework for interactive experiments (Frinex)

All online tests were programmed in Frinex (Withers, 2016). Frinex has been designed and developed by Peter Withers and has been used at the Max Planck Institute for Psycholinguistics for online web experiments since 2015 and for offline field experiments since 2016. It is under active development, which allows for custom features to be added for novel experiment requirements. Stimuli can be shown in Frinex in various forms such as text, audio, or video. Visual stimuli can be animated so that the presentation includes movement on screen. Participant responses can be recorded in a number of ways, for example, by simple button clicks, rating buttons, textual input, recorded audio, or video. Timing data are collected during the response period, which includes the time between key presses when textual input is used. All visual elements in the experiment including stimuli presentation can be customized with CSS (cascading style sheets) or with various predefined styles.

Each of the 35 tests on the IDLaS-NL platform is a stand-alone experiment, with its own URL, and associated with a database that stores the data for that experiment only. Each experiment is implemented such that the stimulus material is downloaded before trial onset and such that response information (including reaction times and wav recordings) is uploaded to the experiment database after each trial. This minimizes the influence of internet bandwidth on the precision of logging stimulus and response events during time-critical periods. Response types and response times are recorded locally, in the browser’s cache. The server uses GDPR-compliant SSL certificates for the data transmission.

The collected data are tagged with study and researcher keys and uploaded to the respective experiment database, which enables the retrieval of specific portions of test data from that database. When selected for a study, individual tests are ‘chained together’ by listing them as ‘steps’ in the URL of the first experiment in the chain. This URL also contains the study and researcher keys, which enables the transmission of both keys from one experiment to the next and the use of both keys for tagging the collected data in each experiment.

### Participant testing environment: The Electron application

In principle, all IDLaS-NL studies and individual experiments can be run in any browser. However, given the ever-changing nature of browsers to accommodate the latest technical advancements, for a standardized test administration we strongly recommend IDLaS-NL users to provide their participants with the dedicated application we make available.

This application was built using the Electron software framework (https://www.electronjs.org/), which is designed to create cross-platform desktop applications using web technology. It includes the Chromium browser engine, which is also used in Google Chrome. Thus, the application is nothing more than a customized browser. The application can be run immediately after downloading (i.e., no separate installation procedure is required) and can simply be moved to the trash once it is no longer needed. Windows and Mac versions are available^1^.

Asking all participants to complete the study in the Electron application has several advantages: First, it ensures that all use the same (version of the) browser. Since we developed all tests for use in the Electron application, all experiment features should therefore work without problems. Second, we customized the application such that, upon start, it takes up the full screen size (address line, tabs, and irrelevant visual features have been removed), which reduces the likelihood of participants running other applications alongside the experiments that may consume the laptop’s processing resources and/or internet bandwidth. Moreover, since the application takes up the full screen, participants are more likely to focus their attention on the tests since switching back and forth between distracting browser tabs is not possible. Finally, although the timing precision of trial events is subject to a number of influences (e.g., operating system, CPU, Anwyl-Irvine et al., 2021; Monen et al., in prep.), using the same browser in all participants eliminates one potential additional source of between-participant variability.

If unsolvable issues prevent running the Electron application on a participant’s computer, the tests may also be carried out in a ‘regular’ browser (preferably Chrome). Instructions for such cases are provided in the PDF that is sent after submitting a study configuration. Throughout all test sessions, participants must maintain an active internet connection.

### Hardware

The majority of tests require responses using the mouse or the keyboard. Speaking and listening tests on the IDLaS-NL platform require participants to use a microphone and headphones (integrated speakers work as well, but are not recommended). To ensure that the microphone works properly, a test in the form of an additional Frinex experiment is automatically added to the beginning of each session that contains a test that requires speaking. These tests are tagged internally and are recognized automatically by the system and no action from the user is required. Before using the microphone in the Electron application, participants must give permission by clicking on the appropriate button in a pop-up dialog. The subsequent microphone test consists of participants naming four written Dutch words in a fixed order. Per word, a recording is made. Next, participants are presented with the just-made recordings, one at a time, in random order and have to click on the word they just heard themselves produce. After recognizing all four words successfully, participants can continue and start the test session; in case less than four words were recognized correctly, the process re-starts and is repeated until successful completion. Should the problem persist, participants need to contact their experimenter. Some potential solutions for solving microphone problems are listed on the FAQ page: https://www.mpi.nl/idlas-nl/faq.

### Data retrieval

As mentioned above, all experimental data are tagged with study and researcher keys for later retrieval of the results. Results can be requested via the graphical user interface for each test individually (log on by providing e-mail address and completing CAPTCHA, provide study and researcher keys and click on ‘Open existing study’). Once per hour, the system checks for which test results have been requested and runs an R script to retrieve and process the requested data for those tests.

For language production tests, all recordings (.wav files) that match study and researcher keys are selected, zipped and made available for download. For all other tests, the data points that match study and researcher keys are downloaded from the Frinex server by the R script and are subsequently pre-processed to yield item-level CSV outputs (i.e., one line per participant per item). For some accuracy-based tests, the item-level output is complemented with aggregated summary scores per participant. All resulting CSV output files are zipped, along with a PDF file listing the stimuli for that experiment and a PDF file providing a legend for the output files column headers, and made available for download. Additionally, the zip files containing recordings or CSV files always contain an irregular CSV file, which lists information about potential problems that occurred during the test and/or data retrieval.

The R script eventually sends out an e-mail (one per test) to the provided e-mail address, containing a link that leads to the zip file of the respective test. Once produced, the download link and the associated zip file are available for seven days.

## A step-by-step guide on how to use IDLaS-NL

**Fig. 1:**
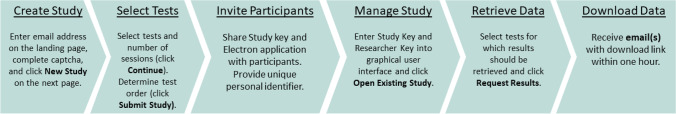
Main steps for creating and running studies using the IDLaS-NL platform

The flowchart in Fig. 1 illustrates the main steps for creating and running studies using the IDLaS-NL platform. Below, we provide more details on each of the steps and give practical recommendations based on our own experience, comprising more than 1000 online participants.

### Registration

The first step to using the IDLaS-NL platform is to register on the landing page. Be sure to use an e-mail address with appropriate security levels. The e-mail address functions as a ‘user name’ and anyone with access to the inbox could get access to the researcher key, which functions as a ‘password’. Users might want to add ‘IDLaS-NL team’ as a secure sender to prevent the e-mail provider from regarding our e-mails as spam.

To advance to the study creation/modification sections of the platform, users must accept the Terms of Use (which includes the Data Processing Agreement; both in the Appendix).

### Creating a study

By clicking on the ‘New study’ button, users initiate the creation of a new study. On the subsequent page, they can select the tests they wish to include in their study. The tests are grouped by the construct they measure. The tests that were not developed and piloted by the IDLaS-NL team, are highlighted as ‘extra tests’. The approximate duration of each test is provided in brackets; clicking on the “i” icon next to each test opens a pop-up featuring a short task description. At the bottom of the page, users can indicate the number of sessions into which they want to divide the tests. A test can only occur once in a session.

Previous research on individual differences has led to practical recommendations (e.g., Miyake et al., 2000), which users may take into account when selecting the tests and the order of tests for their study. As discussed above, when gauging psychological constructs (e.g., processing speed), best practice is to select several tests to address the task impurity problem. Although strongly recommended, this practice is not always feasible (e.g., when testing time is limited). We refer users to Hintz et al. (in prep.) for a discussion of the factor loadings of each test as established in confirmatory factor analyses. The factor loadings give an indication of the test(s) ‘most representative’ of each psychological construct (i.e., has the largest loading), which may inform the choice of which test(s) to include when testing time is limited. Concerning the order of tests, unlike in experiments with factorial designs, studies on individual differences are best run with a fixed test order for all participants. The rationale of this practice is to equate potential influences of the test order on participants’ test performance. It is, advisable to choose an order (i.e., for all participants) that takes effects of fatigue into account (e.g., longer experiments are better administered at the beginning of a test session) and is pleasant for the participants (e.g., tests with similar tasks are best separated by an intervening test with a dissimilar task).

By clicking the ‘Continue’ button, users advance to the page where they can determine the order of tests within their session(s). They can change the number of tests per session by clicking the '- (remove)' button and by clicking the '+ (add another test)' button. The drop-down list next to each test number in a session features all tests selected on the previous page. Users determine the order of tests within a session by entering the tests into positions 1 through n. When satisfied with their configuration, users advance by clicking the ‘Continue’ button. The system then performs a check on the configuration. The following aspects are evaluated:

1. The same test occurs multiple times in one session: Users receive an *error* message and cannot create the study until the error is resolved. They need to adjust their configuration and click on the ‘Continue’ button at the bottom to trigger a new evaluation.
2. The same test occurs in different sessions: Users receive a *warning* message that can be ignored in case this is intentional (click on the ‘Continue’ button).
3. One or more previously selected tests do not occur in any of the sessions: Users receive a *warning* message that can be ignored if this is intentional (click on the ‘Continue’ button).

The study configuration is finalized by clicking the ‘Submit study’ button. Upon clicking that button, the study and researcher keys are generated and stored in the PHP database, along with the provided e-mail address. In more technical terms, a URL for each session is created that contains the included tests as steps, study and the researcher keys and a placeholder for the participant ID. Shortly after submission, an automatic e-mail is sent to the provided address, detailing the study configuration (sessions and tests) and listing study and researcher keys. The e-mail also has a PDF file attachment, which contains, among others, the links where Windows and Mac versions of the Electron application can be downloaded. The server hosting the database uses GDPR-compliant SSL certificates for the data transmission.

### Modifying and removing an existing study

If researchers wish to adjust an existing study configuration, they log on to the landing page by providing their e-mail address and completing the CAPTCHA. Next, within the ‘Existing study’ section, they provide the study key for the study that should be modified and their researcher key and click on ‘Open existing study’. On the following page, users can adjust the study configuration. The graphical interface and configuration evaluation are the same as for creating a new study. The URL(s) as generated and stored when creating the study will be overwritten. Similarly, users may decide to delete their study configuration by clicking the corresponding button on the same page. Note that study deletion refers to deleting the configuration as stored in the PHP database. It does not refer to the deletion of previously collected experimental data, tagged with the corresponding study key.

### Running a study

In order for a participant to take part in a study, they need the following information: (1) the links for downloading the Windows/Mac version of the Electron application, (2) the study key, and (3) a personal identifier that is unique for that participant. This personal identifier can be assigned by the researcher or made up by the participant. The identifier should be at least three characters long and must not contain any personal information. In case of technical issues/questions, users and participants can consult the IDLaS-NL FAQ page: https://www.mpi.nl/idlas-nl/faq.

When inviting participants to take part in a study, users may want to inform them about the duration of the study, the number of sessions, the time that can/should be between two sessions, and the hardware required to complete the study such as headphones and/or a microphone. In case a study consists of multiple sessions, participants need to make sure to enter the same identifier at the beginning of each session.

When taking part in a study, participants open the Electron application. The first page prompts them for the study key and their personal identifier. When clicking on ‘Indienen’ (‘Enter’), the application retrieves the URL, or URLs in case of multiple sessions, associated with the entered study key. The subsequent page lists the sessions the participant still has to complete. Participants need to select the session they want to complete. The tests that are part of the selected session will then be run in succession. At the end of a session, participants are directed to the start screen where study key and personal identifier need to be entered. After entering this information, the remaining sessions are listed and participants select the session to be completed next.

It is important to highlight that closing the application during a test will mark that test as completed. If participants log on again at a later point in time, the application checks whether there is a test in a session that has not been completed yet and will offer to continue from there. For example, let us assume that a study consists of two sessions, which each consist of three tests. A participant closes the application in the middle of test 2 in session 1 and logs on the next day to complete the study by providing the study key and personal identifier. The application will list session 1 and session 2 as incomplete. When selecting session 1, the participant will be presented with test 3 from session 1. When selecting session 2, they will be presented with test 1 from session 2. It is therefore crucial to provide participants with precise information on the time intervals between tests and between sessions and to highlight that once started, a test must be completed in one sitting.

In most studies demographic information about the participants is collected. Given the precise research question, these questionnaires can drastically vary in length. Here, we decided to only collect the most basic information: At the beginning of the first session of each study, participants are asked to provide their age, gender, educational background, native language, handedness, and any medical issues (if applicable *and* if they want to share). Similar to the microphone test, this ‘experiment’ is added automatically to the beginning of the first session. Participants’ (anonymized) responses to the short questionnaire are stored in a database, separate from all other test data. When requesting the results for an experiment from the first session, a separate e-mail is sent containing the results of that questionnaire^2^. Since the short questionnaire only covers basic demographic information, users are invited to complement it using a separate (online) survey.

### Solutions to common technical problems

In spite of our best efforts to debug all components of the IDLaS-NL platform, things can go wrong. We strongly advise researchers to test the battery a number of times, e.g., by sending the link to colleagues using different types of computers. Our own experience has shown that most errors occur (1) during data collection, for example when trying to complete the microphone test but the application has no permission to use the microphone, or the application does have permission, but no speech is recorded and the test cannot be completed. In such cases, it makes sense to test whether the microphone makes recordings at all using a different application. One may also check the operating system settings and manually give permission to the application to use the microphone. The other place where we sometimes see errors is (2) when retrieving results. In such cases, the e-mail that would otherwise contain the link from where the zipped result file can be downloaded features an error message. To give one example, the error message might state that there are no data yet to be retrieved (i.e., no participants have yet finished the specific test). Other error messages may be more complex. When users receive an error message, they should try again at a later point in time. Should the error persist, they may send an e-mail including the provided error message to the developers of IDLaS-NL (idlas-nl@mpi.nl). Please note that we cannot support individual participants experiencing technical issues.

Although we expect them to handle these data with care, it may happen that users lose any of the two keys. In such cases, they need to contact us (idlas-nl@mpi.nl). Our administrators have access to the database that links e-mail addresses to the corresponding study and researcher keys and the study configuration(s). Users will need to provide the e-mail address used for registering their study as well as the experiments included in the study to verify that they are authorized to get access to the keys. Upon positive evaluation, our administrators will send them both keys anew.

### Practical recommendations

Throughout the article, we have already provided some recommendations, however, there are four topics that warrant to be singled out. The first recommendation is a mundane one but may have a substantial influence on the quality of the collected data: Users should be sure to instruct their participants to complete the studies in a quiet environment and on their own. A quiet surrounding will massively improve the quality of speech recordings and will, of course, increase the performance of the participant as compared to a noisy environment.

Second if at all possible participants should complete a study with multiple sessions on the same computer. While the system is capable of handling the same participant completing different sessions on different devices, we do not recommend that–certainly not if tests are included whose dependent variable is time-critical (e.g., auditory lexical decision). The reason is that much of the jitter we see in timing precision of trial events (auditory playback, response logging, Monen et al., in prep.) is associated with features of the hardware that was used (e.g., the keyboard’s polling rate). Using the same computer across different sessions eliminates a potential source of noise, which is crucial for individual-differences studies.

Third, and most importantly, users must pay close attention to the ethical consent procedure. To be clear, we provide the IDLaS-NL web platform as a technical service, but it is the users’ responsibility to ensure that they have sufficient coverage for collecting data using our services. As with other web platforms that can be used for running online experiments, the collected data are stored on our servers. Thus, users must have ethical coverage that approves of storing online experimental data from human participants on European (i.e., Dutch and German) servers. Furthermore, users must implement a way of collecting informed consent (in line with their ethical coverage) from their participants (e.g., a separate online survey, a signed PDF).

## Further information

One of the challenges that experiments conducted via the internet face, compared to conducted in the lab, concerns the timing precision of trial events. In lab studies, the hardware is typically optimized for chronometric experimentation with jitters often below 10 ms across different stimulus delivery and experiment control environments (Bridges et al., 2020). The same timing precision cannot be achieved in online experiments since the experiments are delivered via the internet and run within browsers. Similarly, the hardware the participants use is often inferior to that in the labs. Previous investigations into the jitter of delivering visual stimuli revealed imprecisions in stimulus duration ranging from – 6.24 to 26.02 ms on average (across different platforms, Anwyl-Irvine et al., 2021). The same study found that response logging (RT delay calculated as the difference between known and recorded RT) ranged from 71.33 to 87.40 ms on average (across different platforms).

We conducted a similar study to assess the timing (im)precision in our IDLaS-NL test battery, as programmed in our own online environment (Frinex; Monen et al., in prep.). For six tests that have a time-critical dependent variable, we assessed the jitter in presenting stimuli and recording responses. Overall, the values were comparable to those reported in Anwyl-Irvine et al. (2021). To be precise, we observed that playback of auditory stimuli started approximately 25 ms later than intended and that responses provided via the keyboard were logged approximately 100 ms later than they occurred. It is hard to pinpoint the exact locus of these delays. However, the two most likely sources appear to relate to the browser’s (i.e., the Electron application’s) threading model and the quality of participants’ hardware. A ‘thread’ in computer science refers to the execution of multiple tasks at the same time. Each unit capable of executing code is called a thread. The main thread is the one used by the browser to handle user events, render and paint the display, and to run the majority of the code. ‘Threading model’ refers to the implemented, browser-specific allocation of processing time to the CPU by a task scheduler. Unlike experimental software, such as Presentation, E-Prime or Experiment Builder, browsers do not have a threading model that is optimized for chronometric experimentation, which may contribute to the observed delays. Moreover, in terms of hardware quality, the keyboard’s polling rate and buffer, which relate to the frequency with which the device sends information to the computer are likely to contribute to the delays. Most mice and keyboards used in home environments have a polling rate of 125 Hz, compared to lab devices that often have a polling rate of 1000 or 2000 Hz.

Importantly, our study also showed that these delays were rather consistent across different operating systems (i.e., Windows and Mac), which suggests that, while users have to accept some imprecision in timing, IDLaS-NL may facilitate chronometric experimentation via the internet.

### Concluding comments

IDLAS-NL was developed for Dutch, and the linguistic tasks are consequently suitable only for testing speakers of that language. We are currently developing a German version, and in the near future plan to develop an English version and we would be delighted to hear from any researchers interested in being involved with us in developing these versions and/or versions for other languages.

## Data Availability

There are no data associated with the present manuscript.

## Footnotes

1 Just as other browsers, the Electron application leaves traces on participants’ computers. These traces include timestamps, keys, hashes, and participant responses, and are neither harmful nor considered sensitive information. The FAQ page (https://www.mpi.nl/idlas-nl/faq) provides a simple tutorial for removing the traces after study completion.

2 Since the questionnaire is administered in the first session, its results are only sent when requesting data for an experiment from the first session.
